# Data on European seafood biomass production by country, sectors, and species in 2004–2014 and on ecological characteristics of the main species produced

**DOI:** 10.1016/j.dib.2018.10.095

**Published:** 2018-10-30

**Authors:** Marie-Anne Blanchet, Raul Primicerio, Aslak Smalås, Juliana Arias-Hansen, Michaela Aschan

**Affiliations:** a Norwegian College of Fishery Science, UiT the Arctic University of Norway, 9037 Tromsø, Norway; b Syntesa sp/f, Fyri Oman Brúgv 2, 513 Syðrugøta, Faroe Islands

## Abstract

In this data article, we present the 2004–2014 average European seafood production volume by production sector, country, and species. The production data originates from the Food and Agriculture Organisation of the United Nations (FAO) and covers three production sectors: Marine fisheries, marine aquaculture, and freshwater production. We present the main ecological characteristics of each species produced or harvested. These species characteristics were retrieved from various published sources and include biological sensitivity to harvesting and temperature ranges for the most important species. These indices were weighted by each species production volume in order to produce maps of European country’s color-coded by their overall temperature range, maximum temperature, and biological sensitivity within each production sector.

**Specifications table**

**Table:** 

Subject area	*Aquaculture, Fisheries, Marine biology, Management, Policy*
More specific subject area	*Fisheries and aquaculture management, European Common Fisheries Policy, sustainability, vulnerability, climate change, adaptation*
Type of data	*Table, Figure*
How data was acquired	*The data was acquired from the Food and Agriculture Organisation of the United Nations website - FAO through a custom –made interface* FishStatJ (http://www.fao.org/fishery/statistics/software/fishstatj/en),
	www.fishbase.de*,*www.sealifebase.org*and peer reviewed references (see reference section)*
Data format	*Raw, aggregated, averaged and partially analyzed*
Experimental factors	*The production volume data was acquired from FAO and averaged for the period 2004–2014. The species’ list corresponds to the species contributing to 90% of each country’s 2004–2014 averaged production volume for each production sector.*
Experimental features	*The data consists of the list of European countries and the list of species (English and Latin names) per sector (marine fisheries, marine aquaculture and freshwater production. For each species we provide the average production volume, the biological sensitivity, the temperature preference range (minimum, maximum, mean) and the origin of these data (fishbase.org, sealifebase.org or peer reviewed references)*
Data source location	http://www.fao.org/home/en/
	www.fishbase.de
	www.sealifebase.org
	*peer reviewed references (see reference section)*
Data accessibility	*The data is with this article and is freely and publicly available for any academic, educational and research purposes from UiT, the Arctic University of Norway research data portal*https://dataverse.no/dataset.xhtml?persistentId=doi:10.18710/LI8A7X
	DOI: 10.18710/LI8A7X

**Value of the data**

• Species-specific temperature range and extremes can allow for further studies, for example, what if scenarios.
• Species-specific biological sensitivity allows for comparisons between on-going studies on fish production and coastal communities vulnerability to climate change in Europe and elsewhere.
• The data can be used to provide an indicator of the vulnerability of each European country’s seafood production to climate change based on the country’s production volume and the main species produced or harvested. The main species stand for at least 90% of the production volume in each sector.
• The data provides scientists, policy-makers, and industry actors with a recent overview of the seafood production of the most important species in the European Region and the European Economic Area. It allows comparisons of seafood production vulnerability to climate change of between countries and sectors: Marine fisheries, marine aquaculture, and freshwater production.

## 1 Data

Table 1 provides a list of European countries that produce seafood through their marine fisheries, marine aquaculture, and/or freshwater production sectors. For each country, we provide a list of species (English and Latin names) and their averaged (2004–2014) production volume in tons. The species’ list corresponds to the species contributing to 90% of each country’s 2004–2014 averaged production volume for each production sector. An important addition to the production volume dataset is the temperature range, the mean temperature, and the biological sensitivity [1] of each species harvested by each European country. This allows us to explore the vulnerability to warming of each country by production sector and of each production sector at the European level.

**Fig. 1:**
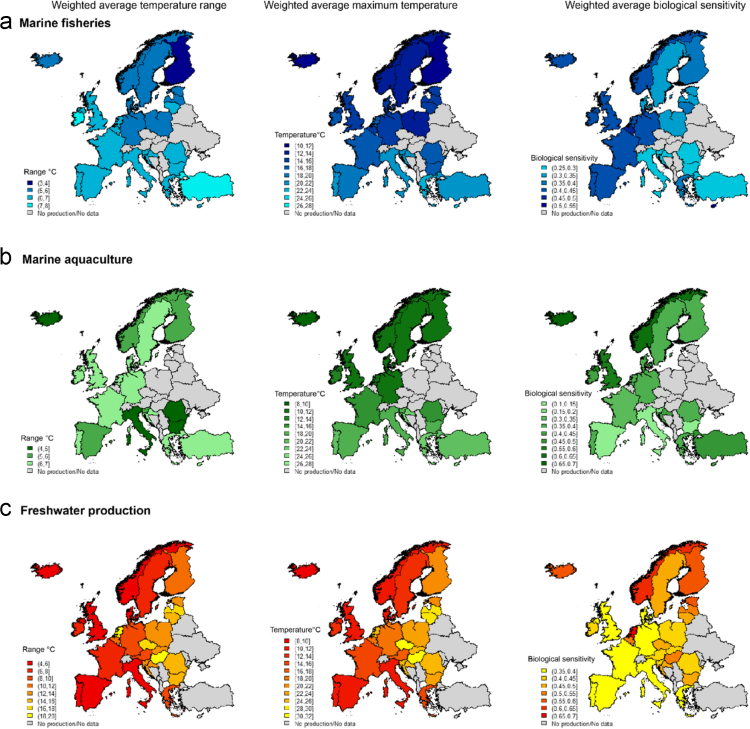
Weighted average temperature range (first image in each row), weighted average maximum temperature Tmax (second image in each row) and weighted average biological sensitivity (third image in each row) for each country based on the species representing 90% of the European production (in volume) between 2004 and 2014 across three production sectors (a) marine fisheries, (b) marine aquaculture, and (c) freshwater production. Darker hues indicate narrower temperature ranges (first image in each row), higher maximum temperature (second image in each row) or higher biological sensitivity index (third image in each row). Note that the scale is different for each production sector in order to keep the maps readable due to large numerical differences across sectors.

Fig. 1 presents an aggregation and weighting of the data from Table 1. It is composed of nine maps of the European Region; each row representing a production sector (a. marine fisheries, b. marine aquaculture, and c. freshwater production) and each column a data type (temperature range, maximum temperature, and biological sensitivity). Darker hues indicate narrower temperature ranges (first image in each row), higher maximum temperature (second image in each row), or higher biological sensitivity index (third image in each row).

## 2 Experimental design, materials, and methods

We define seafood as farmed and captured fish, shellfish and seaweed products from marine and freshwater ecosystems that directly or indirectly (as feed) are meant for human consumption (EU-2020 project ClimeFish Description of Actions www.climefish.eu).

We define Europe as EU-28, EAA (Iceland, Norway, and Lichtenstein), Turkey and the Faroe Islands. Data from the Channel Islands, the Isle of Man, and Gibraltar were included in the UK production statistics; data from Monaco were included in the French production and data from San Marino were included in the Italian production. Production volume data were extracted from the FAO database using the custom-made interface FishStatJ (http://www.fao.org/fishery/statistics/software/fishstatj/en) and averaged over a period of 11 years (2004–2014). Data are presented as an 11-year (2004–2014) average in tons for each species, within each production sector (marine fisheries, marine aquaculture, and freshwater production) for each country (Table 1). Production volume values in tons were averaged in order to smooth out the yearly variations and cope with the poor quality of data for some countries in certain years. Species representing 90% of the production volume for each country were included. A species production volume is only recorded by FAO if it is greater than 0.5 t; therefore, production volumes below 0.5 t are recorded as 0 in Table 1.

Table 1 also includes ecological characteristics of each species such as their biological sensitivity (BS), the temperature range limits (minimum and maximum) and the central tendency in temperature preference (median) that they are currently exposed to (see [9] for details). The temperature range limits (Tmin and Tmax) are the 25th and 75th percentile of the temperature preference. If a species was not included in [1] information was extracted from peer-reviewed literature and the reference is included in Table 1. The BS is based on [2] and extracted from Fishbase [3], or Sealifebase [4]. This index is a combination of ecological and life history traits that influences a species’ sensitivity to removal [2]. In some cases, several species were pooled together by FAO, for example seaweed nei (not elsewhere included) or monkfish nei and are therefore not associated with any temperature or ecological information.

The maps created for Fig. 1 are based on a weighted average of each variable considered (temperature range, maximum temperature, and biological sensitivity). The weighting factor is the production volume for each species within each country. The weighting factor allows to take into account whether a species was harvested in large volumes and thereby would influence more the country or sector vulnerability to warming compared to species harvested in smaller volumes.
