# Supplementary material for: Data on European seafood biomass production by country, sectors, and species in 2004–2014 and on ecological characteristics of the main species produced
Source: Data Brief. 2018 Oct 30;21:1895–9. doi: 10.1016/j.dib.2018.10.095 (PMC6260364; doi:10.1016/j.dib.2018.10.095)
Supplement: Supplementary file 1 — Supplementary material [file mmc1.docx]

**Conflict of interest form Data in Brief DIB – D- 1802418**

*Data article*

We (the authors) declare that we do not have any conflict of interest.

**Title:**

Data on European seafood biomass production by country, sectors and species in 2004-2014 and on ecological characteristics of the main species produced

**Authors:**

Marie-Anne Blanchet^1*^, Raul Primicerio^1^, Aslak Smalås^1^, Juliana Arias-Hansen^2^, Michaela Aschan^1^

**Affiliations:**

^1^Norwegian College of Fishery Science, UiT the Arctic University of Norway, 9037 Tromsø, Norway

^2^ Syntesa sp/f, Fyri Oman Brúgv 2, 513 Syðrugøta, Faroe Islands

**Contact email:**

*Corresponding author: tel: +47 77 72 32 55; e-mail:marie-anne.e.blanchet@uit.no*
