# Supplementary material for: Data on European seafood biomass production by country, sectors, and species in 2004–2014 and on ecological characteristics of the main species produced
Source: Data Brief. 2018 Oct 30;21:1895–9. doi: 10.1016/j.dib.2018.10.095 (PMC6260364; doi:10.1016/j.dib.2018.10.095)
Supplement: Supplementary file 2 — Supplementary material [file mmc2.docx]

Table 1: Seafood species contributing to 90% of each country’s production in volume per sector for the period 2004-2014. The average production volume is in tons. The sector column refers to M= marine fishery, A= marine aquaculture and F=freshwater. “nei” means “not included elsewhere” for species with a general name (ex: monkfish). NA means that the information was not available. The temperature range limits (Tmin and Tmax) are the 25^th^ and 75^th^ percentile of the temperature preference respectively while the central tendency in temperature preference (Tmed) is the 50^th^ percentile, based on Cheung et al. 2013. The biological sensitivity is based on Cheung et al. (2005) and extracted from Fishbase or Sealifebase.

| **Country** | **Species common name** | **Latin name** | **Average (t)** | **BS** | **Tmin** | **Tmax** | **Tmed** | **source** | **Sector** |
| --- | --- | --- | --- | --- | --- | --- | --- | --- | --- |
| Belgium | European plaice | *Pleuronectes platessus* | 6140 | 0.71 | 9 | 15 | 12 | Cheung et al 2013 | M |
| Belgium | Common sole | *Solea solea* | 3789 | 0.36 | 12 | 19 | 16 | Cheung et al 2013 | M |
| Belgium | Atlantic cod | *Gadus morhua* | 1341 | 0.65 | 3 | 9 | 6 | Cheung et al 2013 | M |
| Belgium | Monkfishes nei | *non specific* | 1257 | NA | NA | NA | NA | NA | M |
| Belgium | Common shrimp | *Crangon crangon* | 1088 | 0.10 | 9 | 14 | 11 | Cheung et al 2013 | M |
| Belgium | Lemon sole | *Microstomus kitt* | 962 | 0.34 | 3 | 10 | 6 | Cheung et al 2013 | M |
| Belgium | Rays and skates nei | *non specific* | 904 | NA | NA | NA | NA | NA | M |
| Belgium | Great Atlantic scallop | *Pecten maximus* | 752 | 0.26 | 11 | 15 | 12 | Cheung et al 2013 | M |
| Belgium | Common cuttlefish | *Sepia officinalis* | 657 | 0.30 | 15 | 23 | 19 | Cheung et al 2013 | M |
| Belgium | Common dab | *Limanda limanda* | 573 | 0.26 | 9 | 12 | 10 | Cheung et al 2013 | M |
| Belgium | Tub gurnard | *Chelidonichthys lucerna* | 565 | 0.40 | 8 | 22 | 15 | Cheung et al 2013 | M |
| Belgium | Dogfishes and hounds nei | *non specific* | 499 | NA | NA | NA | NA | NA | M |
| Belgium | Pouting(=Bib) | *Trisopterus luscus* | 430 | 0.44 | 10 | 14 | 12 | Cheung et al 2013 | M |
| Belgium | Turbot | *Scophthalmus maximus* | 389 | 0.43 | 9 | 14 | 11 | Cheung et al 2013 | M |
| Belgium | Brill | *Scophthalmus rhombus* | 359 | 0.32 | 7 | 14 | 11 | Cheung et al 2013 | M |
| Belgium | European flounder | *Platichthys flesus* | 339 | 0.42 | 10 | 15 | 12 | Cheung et al 2013 | M |
| Belgium | Whiting | *Merlangius merlangus* | 323 | 0.37 | 9 | 14 | 11 | Cheung et al 2013 | M |
| Belgium | Thornback ray | *Raja clavata* | 317 | 0.72 | 14 | 20 | 17 | Cheung et al 2013 | M |
| Belgium | Haddock | *Melanogrammus aeglefinus* | 303 | 0.47 | 5 | 10 | 7 | Cheung et al 2013 | M |
| Belgium | Red gurnard | *Chelidonichthys cuculus* | 293 | 0.45 | NA | NA | NA | Fishbase | M |
| Belgium | Megrim | *Lepidorhombus whiffiagonis* | 275 | 0.62 | 12 | 19 | 16 | Cheung et al 2013 | M |
| Bulgaria | European sprat | *Sprattus sprattus* | 3352 | 0.25 | 9 | 16 | 12 | Cheung et al 2013 | M |
| Bulgaria | Sea snails | *non specific* | 3311 | NA | NA | NA | NA | NA | M |
| Bulgaria | Mediterranean horse mackerel | *Trachurus mediterraneus* | 179 | 0.47 | 18 | 20 | 19 | Cheung et al 2013 | M |
| Croatia | European pilchard(=Sardine) | *Sardina pilchardus* | 31670 | 0.27 | 13 | 19 | 16 | Cheung et al 2013 | M |
| Croatia | European anchovy | *Engraulis encrasicolus* | 11228 | 0.24 | 15 | 23 | 21 | Cheung et al 2013 | M |
| Croatia | Marine fishes nei | *non specific* | 3590 | NA | NA | NA | NA | NA | M |
| Croatia | European hake | *Merluccius merluccius* | 857 | 0.64 | 15 | 20 | 18 | Cheung et al 2013 | M |
| Croatia | Atlantic bluefin tuna | *Thunnus thynnus* | 642 | 0.82 | 19 | 27 | 24 | Cheung et al 2013 | M |
| Croatia | Red mullet | *Mullus barbatus* | 573 | 0.29 | 12 | 22 | 17 | Cheung et al 2013 | M |
| Cyprus | Albacore | *Thunnus alalunga* | 356 | 0.58 | 20 | 26 | 23 | Cheung et al 2013 | M |
| Cyprus | Bogue | *Boops boops* | 191 | 0.41 | 16 | 24 | 20 | Cheung et al 2013 | M |
| Cyprus | Picarels nei | *non specific* | 189 | NA | NA | NA | NA | NA | M |
| Cyprus | Marine fishes nei | *non specific* | 139 | NA | NA | NA | NA | NA | M |
| Cyprus | Surmullet | *Mullus surmuletus* | 81 | 0.39 | 13 | 23 | 19 | Cheung et al 2013 | M |
| Cyprus | Octopuses, etc. nei | *non specific* | 78 | NA | NA | NA | NA | NA | M |
| Cyprus | Atlantic bluefin tuna | *Thunnus thynnus* | 51 | 0.82 | 19 | 27 | 24 | Cheung et al 2013 | M |
| Cyprus | Swordfish | *Xiphias gladius* | 47 | 0.72 | 23 | 27 | 26 | Cheung et al 2013 | M |
| Cyprus | Common cuttlefish | *Sepia officinalis* | 41 | 0.30 | 15 | 23 | 19 | Cheung et al 2013 | M |
| Cyprus | Red mullet | *Mullus barbatus* | 38 | 0.29 | 12 | 22 | 17 | Cheung et al 2013 | M |
| Cyprus | Parrotfish | *Sparisoma cretense* | 37 | 0.36 | NA | NA | 26 | Fishbase | M |
| Cyprus | Spinefeet(=Rabbitfishes) nei | *non specific* | 36 | NA | NA | NA | NA | NA | M |
| Cyprus | Blotched picarel | *Spicara maena* | 25 | 0.45 | 12 | 19 | 16 | Cheung et al 2013 | M |
| Cyprus | Sargo breams nei | *non specific* | 25 | NA | NA | NA | NA | NA | M |
| Cyprus | European squid | *Loligo vulgaris vulgaris* | 21 | 0.30 | NA | NA | NA | Sealifebase | M |
| Cyprus | Axillary seabream | *Pagellus acarne* | 19 | 0.43 | 18 | 20 | 19 | Cheung et al 2013 | M |
| Cyprus | Common pandora | *Pagellus erythrinus* | 18 | 0.40 | 16 | 20 | 18 | Cheung et al 2013 | M |
| Cyprus | Comber | *Serranus cabrilla* | 16 | 0.36 | 20 | 27 | 23 | Cheung et al 2013 | M |
| Cyprus | European hake | *Merluccius merluccius* | 16 | 0.64 | 15 | 20 | 18 | Cheung et al 2013 | M |
| Cyprus | Scorpionfishes nei | *non specific* | 16 | NA | NA | NA | NA | NA | M |
| Cyprus | Red porgy | *Pagrus pagrus* | 14 | 0.66 | 21 | 27 | 25 | Cheung et al 2013 | M |
| Denmark | Sandeels(=Sandlances) nei | *Ammodytes tobianus* | 222004 | 0.23 | 11 | 16 | 13 | Cheung et al 2013 | M |
| Denmark | European sprat | *Sprattus sprattus* | 181743 | 0.25 | 9 | 16 | 12 | Cheung et al 2013 | M |
| Denmark | Atlantic herring | *Clupea harengus* | 120654 | 0.39 | 8 | 11 | 5 | Cheung et al 2013 | M |
| Denmark | Blue mussel | *Mytilus edulis* | 48538 | 0.36 | 4 | 11 | 8 | Cheung et al 2013 | M |
| Denmark | Atlantic mackerel | *Scomber scombrus* | 30531 | 0.44 | 8 | 16 | 12 | Cheung et al 2013 | M |
| Denmark | Blue whiting(=Poutassou) | *Micromesistius poutassou* | 26846 | 0.33 | 10 | 19 | 15 | Cheung et al 2013 | M |
| Denmark | Atlantic cod | *Gadus morhua* | 25956 | 0.65 | 3 | 9 | 6 | Cheung et al 2013 | M |
| Denmark | Norway pout | *Trisopterus esmarkii* | 24895 | 0.26 | 8 | 11 | 9 | Cheung et al 2013 | M |
| Denmark | European plaice | *Pleuronectes platessus* | 19429 | 0.71 | 9 | 15 | 12 | Cheung et al 2013 | M |
| Estonia | European sprat | *Sprattus sprattus* | 41364 | 0.25 | 9 | 16 | 12 | Cheung et al 2013 | M |
| Estonia | Atlantic herring | *Clupea harengus* | 25916 | 0.39 | 8 | 11 | 5 | Cheung et al 2013 | M |
| Faroe Islands | Blue whiting(=Poutassou) | *Micromesistius poutassou* | 175053 | 0.33 | 10 | 19 | 15 | Cheung et al 2013 | M |
| Faroe Islands | Atlantic herring | *Clupea harengus* | 72797 | 0.39 | 8 | 11 | 5 | Cheung et al 2013 | M |
| Faroe Islands | Atlantic mackerel | *Scomber scombrus* | 61174 | 0.44 | 8 | 16 | 12 | Cheung et al 2013 | M |
| Faroe Islands | Saithe(=Pollock) | *Pollachius virens* | 52295 | 0.59 | 4 | 11 | 6 | Cheung et al 2013 | M |
| Faroe Islands | Atlantic cod | *Gadus morhua* | 31207 | 0.65 | 3 | 9 | 6 | Cheung et al 2013 | M |
| Faroe Islands | Capelin | *Mallotus villosus* | 18720 | 0.23 | 1 | 5 | 3 | Cheung et al 2013 | M |
| Faroe Islands | Argentines | *Argentina sphyraena* | 13486 | 0.36 | 12 | 19 | 16 | Cheung et al 2013 | M |
| Faroe Islands | Haddock | *Melanogrammus aeglefinus* | 12770 | 0.47 | 5 | 10 | 7 | Cheung et al 2013 | M |
| Finland | Atlantic herring | *Clupea harengus* | 94904 | 0.39 | 8 | 11 | 5 | Cheung et al 2013 | M |
| Finland | European sprat | *Sprattus sprattus* | 17988 | 0.25 | 9 | 16 | 12 | Cheung et al 2013 | M |
| France | European pilchard(=Sardine) | *Sardina pilchardus* | 31034 | 0.27 | 13 | 19 | 16 | Cheung et al 2013 | M |
| France | Tangle | *Laminaria digitata* | 26833 | NA | NA | 22 | 10 | Bolton and Lüning 1982 | M |
| France | Great Atlantic scallop | *Pecten maximus* | 25792 | 0.26 | 11 | 15 | 12 | Cheung et al 2013 | M |
| France | Atlantic herring | *Clupea harengus* | 24352 | 0.39 | 8 | 11 | 5 | Cheung et al 2013 | M |
| France | European hake | *Merluccius merluccius* | 21118 | 0.64 | 15 | 20 | 18 | Cheung et al 2013 | M |
| France | Monkfishes nei | *non specific* | 20434 | NA | NA | NA | NA | NA | M |
| France | Atlantic mackerel | *Scomber scombrus* | 18019 | 0.44 | 8 | 16 | 12 | Cheung et al 2013 | M |
| France | Saithe(=Pollock) | *Pollachius virens* | 15329 | 0.59 | 4 | 11 | 6 | Cheung et al 2013 | M |
| France | Whelk | *Buccinum undatum* | 12327 | NA | 10 | 12 | 11 | Cheung et al 2013 | M |
| France | Whiting | *Merlangius merlangus* | 12081 | 0.37 | 9 | 14 | 11 | Cheung et al 2013 | M |
| France | Blue whiting(=Poutassou) | *Micromesistius poutassou* | 11769 | 0.33 | 10 | 19 | 15 | Cheung et al 2013 | M |
| France | Atlantic horse mackerel | *Trachurus trachurus* | 9213 | 0.59 | 14 | 22 | 18 | Cheung et al 2013 | M |
| France | Atlantic cod | *Gadus morhua* | 8346 | 0.65 | 3 | 9 | 6 | Cheung et al 2013 | M |
| France | Common sole | *Solea solea* | 8083 | 0.36 | 12 | 19 | 16 | Cheung et al 2013 | M |
| France | North European kelp | *non specific* | 7264 | NA | NA | NA | NA | NA | M |
| France | Cuttlefish, bobtail squids nei | *non specific* | 7202 | 0.10 | NA | NA | NA | NA | M |
| France | Haddock | *Melanogrammus aeglefinus* | 7085 | 0.47 | 5 | 10 | 7 | Cheung et al 2013 | M |
| France | European anchovy | *Engraulis encrasicolus* | 6403 | 0.24 | 15 | 23 | 21 | Cheung et al 2013 | M |
| France | Common cuttlefish | *Sepia officinalis* | 6337 | 0.30 | 15 | 23 | 19 | Cheung et al 2013 | M |
| France | Edible crab | *Cancer pagurus* | 5686 | 0.10 | 11 | 16 | 14 | Cheung et al 2013 | M |
| France | Small-spotted catshark | *Scyliorhinus canicula* | 5418 | 0.62 | 10 | 22 | 17 | Cheung et al 2013 | M |
| France | Various squids nei | *European common squid* | 5258 | 0.10 | NA | NA | NA | Sealifebase | M |
| France | European seabass | *Dicentrarchus labrax* | 5125 | 0.49 | 10 | 17 | 14 | Cheung et al 2013 | M |
| France | Pouting(=Bib) | *Trisopterus luscus* | 4979 | 0.44 | 10 | 14 | 12 | Cheung et al 2013 | M |
| France | European conger | *Conger conger* | 4952 | 0.86 | 9 | 22 | 16 | Cheung et al 2013 | M |
| France | Norway lobster | *Nephrops norvegicus* | 4881 | 0.14 | 14 | 20 | 17 | Cheung et al 2013 | M |
| France | Atlantic bluefin tuna | *Thunnus thynnus* | 4657 | 0.82 | 19 | 27 | 24 | Cheung et al 2013 | M |
| France | Seaweeds nei | *non specific* | 4483 | NA | NA | NA | NA | NA | M |
| France | Spinous spider crab | *Maja squinado* | 4322 | 0.12 | 17 | 23 | 20 | Cheung et al 2013 | M |
| France | Common European bittersweet | *Glycymeris glycymeris* | 4183 | NA | NA | NA | 11 | Sealifebase | M |
| France | Red gurnard | *Chelidonichthys cuculus* | 4135 | 0.45 | NA | NA | NA | Fishbase | M |
| France | Albacore | *Thunnus alalunga* | 3903 | 0.58 | 20 | 26 | 23 | Cheung et al 2013 | M |
| France | Blue mussel | *Mytilus edulis* | 3663 | 0.36 | 4 | 11 | 8 | Cheung et al 2013 | M |
| France | Black seabream | *Spondyliosoma cantharus* | 3472 | 0.37 | 14 | 23 | 19 | Cheung et al 2013 | M |
| France | Megrim | *Lepidorhombus whiffiagonis* | 3395 | 0.62 | 12 | 19 | 16 | Cheung et al 2013 | M |
| France | Pollack | *Pollachius pollachius* | 3236 | 0.59 | 9 | 12 | 10 | Cheung et al 2013 | M |
| France | Surmullet | *Mullus surmuletus* | 3182 | 0.39 | 13 | 23 | 19 | Cheung et al 2013 | M |
| France | Queen scallop | *Chlamys opercularis* | 3135 | 0.22 | 11 | 13 | 12 | Cheung et al 2013 | M |
| France | European plaice | *Pleuronectes platessus* | 2964 | 0.71 | 9 | 15 | 12 | Cheung et al 2013 | M |
| France | Smooth-hounds nei | *non specific* | 2814 | NA | NA | NA | NA | NA | M |
| France | Roundnose grenadier | *Coryphaenoides rupestris* | 2742 | 0.67 | 6 | 13 | 10 | Cheung et al 2013 | M |
| France | Blue ling | *Molva dypterygia* | 2589 | 0.75 | 5 | 17 | 11 | Cheung et al 2013 | M |
| France | Cuckoo ray | *Leucoraja naevus* | 2478 | 0.62 | 11 | 22 | 17 | Cheung et al 2013 | M |
| France | Black scabbardfish | *Aphanopus carbo* | 2382 | 0.64 | 7 | 15 | 11 | Cheung et al 2013 | M |
| France | Ling | *Molva molva* | 2193 | 0.77 | 10 | 17 | 14 | Cheung et al 2013 | M |
| Germany | Atlantic herring | *Clupea harengus* | 57122 | 0.39 | 8 | 11 | 5 | Cheung et al 2013 | M |
| Germany | European sprat | *Sprattus sprattus* | 22587 | 0.25 | 9 | 16 | 12 | Cheung et al 2013 | M |
| Germany | Atlantic mackerel | *Scomber scombrus* | 20619 | 0.44 | 8 | 16 | 12 | Cheung et al 2013 | M |
| Germany | Atlantic horse mackerel | *Trachurus trachurus* | 18697 | 0.59 | 14 | 22 | 18 | Cheung et al 2013 | M |
| Germany | Common shrimp | *Crangon crangon* | 17779 | 0.10 | 9 | 14 | 11 | Cheung et al 2013 | M |
| Germany | Blue whiting(=Poutassou) | *Micromesistius poutassou* | 17366 | 0.33 | 10 | 19 | 15 | Cheung et al 2013 | M |
| Germany | Atlantic cod | *Gadus morhua* | 16974 | 0.65 | 3 | 9 | 6 | Cheung et al 2013 | M |
| Germany | Saithe(=Pollock) | *Pollachius virens* | 13412 | 0.59 | 4 | 11 | 6 | Cheung et al 2013 | M |
| Germany | Sandeels(=Sandlances) nei | *Ammodytes tobianus* | 5669 | 0.23 | 11 | 16 | 13 | Cheung et al 2013 | M |
| Germany | Greenland halibut | *Reinhardtius hippoglossoides* | 4807 | 0.70 | 4 | 9 | 6 | Cheung et al 2013 | M |
| Greece | European anchovy | *Engraulis encrasicolus* | 12220 | 0.24 | 15 | 23 | 21 | Cheung et al 2013 | M |
| Greece | European pilchard(=Sardine) | *Sardina pilchardus* | 8582 | 0.27 | 13 | 19 | 16 | Cheung et al 2013 | M |
| Greece | Marine fishes nei | *non specific* | 8090 | NA | NA | NA | NA | NA | M |
| Greece | European hake | *Merluccius merluccius* | 4479 | 0.64 | 15 | 20 | 18 | Cheung et al 2013 | M |
| Greece | Bogue | *Boops boops* | 3584 | 0.41 | 16 | 24 | 20 | Cheung et al 2013 | M |
| Greece | Picarels nei | *non specific* | 2584 | NA | NA | NA | NA | NA | M |
| Greece | Mediterranean horse mackerel | *Trachurus mediterraneus* | 2564 | 0.47 | 18 | 20 | 19 | Cheung et al 2013 | M |
| Greece | Chub mackerel | *Scomber japonicus* | 2421 | 0.31 | 18 | 27 | 23 | Cheung et al 2013 | M |
| Greece | Caramote prawn | *Penaeus kerathurus* | 2305 | 0.17 | 20 | 26 | 23 | Cheung et al 2013 | M |
| Greece | Common octopus | *Octopus vulgaris* | 1899 | 0.78 | 23 | 26 | 28 | Cheung et al 2013 | M |
| Greece | Red mullet | *Mullus barbatus* | 1892 | 0.29 | 12 | 22 | 17 | Cheung et al 2013 | M |
| Greece | Shads nei | *non specific* | 1868 | NA | NA | NA | NA | NA | M |
| Greece | Flathead grey mullet | *Mugil cephalus* | 1599 | 0.50 | 25 | 28 | 27 | Cheung et al 2013 | M |
| Greece | Common cuttlefish | *Sepia officinalis* | 1514 | 0.30 | 15 | 23 | 19 | Cheung et al 2013 | M |
| Greece | Swordfish | *Xiphias gladius* | 1337 | 0.72 | 23 | 27 | 26 | Cheung et al 2013 | M |
| Greece | Surmullet | *Mullus surmuletus* | 1278 | 0.39 | 13 | 23 | 19 | Cheung et al 2013 | M |
| Greece | Various squids nei | *European common squid* | 1162 | 0.10 | NA | NA | NA | Sealifebase | M |
| Greece | Natantian decapods nei | *non specific* | 1125 | NA | NA | NA | NA | NA | M |
| Greece | Monkfishes nei | *non specific* | 1016 | NA | NA | NA | NA | NA | M |
| Greece | Blue whiting(=Poutassou) | *Trachurus mediterraneus* | 937 | 0.33 | 10 | 19 | 15 | Cheung et al 2013 | M |
| Greece | Atlantic bonito | *Sarda sarda* | 858 | 0.33 | 18 | 26 | 23 | Cheung et al 2013 | M |
| Greece | Common sole | *Solea solea* | 772 | 0.36 | 12 | 19 | 16 | Cheung et al 2013 | M |
| Greece | Common squids nei | *Loligo vulgaris* | 703 | 0.19 | 17 | 19 | 18 | Cheung et al 2013 | M |
| Greece | Octopuses, etc. nei | *non specific* | 679 | NA | NA | NA | NA | NA | M |
| Greece | Pandoras nei | *non specific* | 533 | NA | NA | NA | NA | NA | M |
| Greece | European seabass | *Dicentrarchus labrax* | 524 | 0.49 | 10 | 17 | 14 | Cheung et al 2013 | M |
| Greece | European conger | *Conger conger* | 512 | 0.86 | 9 | 22 | 16 | Cheung et al 2013 | M |
| Greece | Scorpionfishes nei | *non specific* | 504 | NA | NA | NA | NA | NA | M |
| Greece | Large-eye dentex | *Dentex macrophthalmus* | 483 | 0.33 | 20 | 25 | 23 | Cheung et al 2013 | M |
| Greece | Red porgy | *Pagrus pagrus* | 413 | 0.66 | 21 | 27 | 25 | Cheung et al 2013 | M |
| Greece | Norway lobster | *Nephrops norvegicus* | 401 | 0.14 | 14 | 20 | 17 | Cheung et al 2013 | M |
| Iceland | Capelin | *Mallotus villosus* | 305926 | 0.23 | 1 | 5 | 3 | Cheung et al 2013 | M |
| Iceland | Atlantic herring | *Clupea harengus* | 243897 | 0.39 | 8 | 11 | 5 | Cheung et al 2013 | M |
| Iceland | Atlantic cod | *Gadus morhua* | 199385 | 0.65 | 3 | 9 | 6 | Cheung et al 2013 | M |
| Iceland | Blue whiting(=Poutassou) | *Micromesistius poutassou* | 179015 | 0.33 | 10 | 19 | 15 | Cheung et al 2013 | M |
| Iceland | Atlantic mackerel | *Scomber scombrus* | 93052 | 0.44 | 8 | 16 | 12 | Cheung et al 2013 | M |
| Iceland | Haddock | *Melanogrammus aeglefinus* | 74237 | 0.47 | 5 | 10 | 7 | Cheung et al 2013 | M |
| Iceland | Saithe(=Pollock) | *Pollachius virens* | 60061 | 0.59 | 4 | 11 | 6 | Cheung et al 2013 | M |
| Iceland | Golden redfish | *Sebastes norvegicus* | 44344 | 0.71 | 3 | 7 | 5 | Cheung et al 2013 | M |
| Iceland | Beaked redfish | *Sebastes mentella* | 16954 | 0.56 | 2 | 7 | 4 | Fishbase | M |
| Ireland | Atlantic mackerel | *Scomber scombrus* | 58654 | 0.44 | 8 | 16 | 12 | Cheung et al 2013 | M |
| Ireland | Atlantic horse mackerel | *Trachurus trachurus* | 35744 | 0.59 | 14 | 22 | 18 | Cheung et al 2013 | M |
| Ireland | Boarfish | *Capros aper* | 32993 | 0.51 | 12 | 23 | 19 | Cheung et al 2013 | M |
| Ireland | North Atlantic rockweed | *non specific* | 28018 | NA | NA | NA | NA | NA | M |
| Ireland | Atlantic herring | *Clupea harengus* | 27176 | 0.39 | 8 | 11 | 5 | Cheung et al 2013 | M |
| Ireland | Blue whiting(=Poutassou) | *Micromesistius poutassou* | 27085 | 0.33 | 10 | 19 | 15 | Cheung et al 2013 | M |
| Ireland | Norway lobster | *Nephrops norvegicus* | 8132 | 0.14 | 14 | 20 | 17 | Cheung et al 2013 | M |
| Ireland | Edible crab | *Cancer pagurus* | 8015 | 0.10 | 11 | 16 | 14 | Cheung et al 2013 | M |
| Ireland | Whiting | *Merlangius merlangus* | 5097 | 0.37 | 9 | 14 | 11 | Cheung et al 2013 | M |
| Ireland | European sprat | *Sprattus sprattus* | 4247 | 0.25 | 9 | 16 | 12 | Cheung et al 2013 | M |
| Ireland | Haddock | *Melanogrammus aeglefinus* | 3591 | 0.47 | 5 | 10 | 7 | Cheung et al 2013 | M |
| Ireland | Angler(=Monk) | *Lophius piscatorius* | 3419 | 0.72 | 10 | 17 | 14 | Cheung et al 2013 | M |
| Ireland | Whelk | *Buccinum undatum* | 2892 | NA | 10 | 12 | 11 | Cheung et al 2013 | M |
| Italy | European anchovy | *Engraulis encrasicolus* | 51643 | 0.24 | 15 | 23 | 21 | Cheung et al 2013 | M |
| Italy | Striped venus | *Venus gallina* | 21011 | 0.10 | 12 | 17 | 14 | Cheung et al 2013 | M |
| Italy | European pilchard(=Sardine) | *Sardina pilchardus* | 16261 | 0.27 | 13 | 19 | 16 | Cheung et al 2013 | M |
| Italy | European hake | *Merluccius merluccius* | 12191 | 0.64 | 15 | 20 | 18 | Cheung et al 2013 | M |
| Italy | Marine fishes nei | *non specific* | 10754 | NA | NA | NA | NA | NA | M |
| Italy | Deep-water rose shrimp | *Parapenaeus longirostris* | 9403 | 0.10 | 23 | 27 | 25 | Cheung et al 2013 | M |
| Italy | Cuttlefish, bobtail squids nei | *non specific* | 8035 | 0.10 | NA | NA | NA | NA | M |
| Italy | Red mullet | *Mullus barbatus* | 6278 | 0.29 | 12 | 22 | 17 | Cheung et al 2013 | M |
| Italy | Spottail mantis squillid | *Squilla mantis* | 5982 | 0.10 | 19 | 25 | 22 | Cheung et al 2013 | M |
| Italy | Horned and musky octopuses | *Eledone cirrhosa* | 5712 | 0.30 | 16 | 20 | 18 | Cheung et al 2013 | M |
| Italy | Swordfish | *Xiphias gladius* | 5476 | 0.72 | 23 | 27 | 26 | Cheung et al 2013 | M |
| Italy | Jack and horse mackerels nei | *Trachurus mediterraneus* | 4116 | 0.47 | 18 | 20 | 19 | Cheung et al 2013 | M |
| Italy | Gastropods nei | *non specific* | 3872 | NA | NA | NA | NA | NA | M |
| Italy | Common octopus | *Octopus vulgaris* | 3582 | 0.78 | 23 | 26 | 28 | Cheung et al 2013 | M |
| Italy | Broadtail shortfin squid | *Illex coindetii* | 3421 | NA | 18 | 25 | 22 | Cheung et al 2013 | M |
| Italy | Mullets nei | *non specific* | 3384 | NA | NA | NA | NA | NA | M |
| Italy | Mediterranean mussel | *Mytilus galloprovincialis* | 3188 | 0.10 | 14 | 19 | 15 | Cheung et al 2013 | M |
| Italy | Norway lobster | *Nephrops norvegicus* | 3145 | 0.14 | 14 | 20 | 17 | Cheung et al 2013 | M |
| Italy | Atlantic bluefin tuna | *Thunnus thynnus* | 2946 | 0.82 | 19 | 27 | 24 | Cheung et al 2013 | M |
| Italy | Aristeid shrimps nei | *Aristeus antennatus* | 2942 | NA | 19 | 20 | 19 | Sealifebase | M |
| Italy | Surmullet | *Mullus surmuletus* | 2492 | 0.39 | 13 | 23 | 19 | Cheung et al 2013 | M |
| Italy | Albacore | *Thunnus alalunga* | 2367 | 0.58 | 20 | 26 | 23 | Cheung et al 2013 | M |
| Italy | Bogue | *Boops boops* | 2365 | 0.41 | 16 | 24 | 20 | Cheung et al 2013 | M |
| Italy | Common sole | *Solea solea* | 2152 | 0.36 | 12 | 19 | 16 | Cheung et al 2013 | M |
| Italy | Silver scabbardfish | *Lepidopus caudatus* | 2105 | 0.53 | 19 | 27 | 24 | Cheung et al 2013 | M |
| Italy | Common squids nei | *Loligo vulgaris* | 1937 | 0.19 | 17 | 19 | 18 | Cheung et al 2013 | M |
| Italy | Monkfishes nei | *non specific* | 1776 | NA | NA | NA | NA | NA | M |
| Italy | Smooth callista | *Callista chione* | 1642 | 0.10 | 10 | 14 | 12 | Cheung et al 2013 | M |
| Italy | Pandoras nei | *non specific* | 1549 | NA | NA | NA | NA | NA | M |
| Italy | Common dolphinfish | *Coryphaena hippurus* | 1546 | 0.40 | 26 | 28 | 27 | Cheung et al 2013 | M |
| Italy | Gurnards, searobins nei | *non specific* | 1540 | NA | NA | NA | NA | NA | M |
| Italy | Marine molluscs nei | *non specific* | 1522 | NA | NA | NA | NA | NA | M |
| Italy | Picarels nei | *non specific* | 1470 | NA | NA | NA | NA | NA | M |
| Italy | Atlantic mackerel | *Scomber scombrus* | 1343 | 0.44 | 8 | 16 | 12 | Cheung et al 2013 | M |
| Italy | Atlantic bonito | *Sarda sarda* | 1269 | 0.33 | 18 | 26 | 23 | Cheung et al 2013 | M |
| Latvia | European sprat | *Sprattus sprattus* | 46643 | 0.25 | 9 | 16 | 12 | Cheung et al 2013 | M |
| Latvia | Atlantic herring | *Clupea harengus* | 22033 | 0.39 | 8 | 11 | 5 | Cheung et al 2013 | M |
| Latvia | Atlantic cod | *Gadus morhua* | 4135 | 0.65 | 3 | 9 | 6 | Cheung et al 2013 | M |
| Lithuania | European sprat | *Sprattus sprattus* | 12220 | 0.25 | 9 | 16 | 12 | Cheung et al 2013 | M |
| Lithuania | Atlantic herring | *Clupea harengus* | 3189 | 0.39 | 8 | 11 | 5 | Cheung et al 2013 | M |
| Lithuania | Atlantic cod | *Gadus morhua* | 2690 | 0.65 | 3 | 9 | 6 | Cheung et al 2013 | M |
| Lithuania | Atlantic horse mackerel | *Trachurus trachurus* | 2376 | 0.59 | 14 | 22 | 18 | Cheung et al 2013 | M |
| Lithuania | Blue whiting(=Poutassou) | *Micromesistius poutassou* | 2221 | 0.33 | 10 | 19 | 15 | Cheung et al 2013 | M |
| Lithuania | Atlantic redfishes nei | *Sebastes marinus* | 1998 | 0.56 | 3 | 6 | 5 | Cheung et al 2013 | M |
| Lithuania | Northern prawn | *Pandalus borealis* | 887 | 0.10 | 3 | 9 | 5 | Cheung et al 2013 | M |
| Lithuania | Atlantic mackerel | *Scomber scombrus* | 878 | 0.44 | 8 | 16 | 12 | Cheung et al 2013 | M |
| Malta | Common dolphinfish | *Coryphaena hippurus* | 382 | 0.40 | 26 | 28 | 27 | Cheung et al 2013 | M |
| Malta | Common dolphinfish | *Coryphaena hippurus* | 382 | 0.40 | 26 | 28 | 27 | Cheung et al 2013 | M |
| Malta | Swordfish | *Xiphias gladius* | 348 | 0.72 | 23 | 27 | 26 | Cheung et al 2013 | M |
| Malta | Swordfish | *Xiphias gladius* | 348 | 0.72 | 23 | 27 | 26 | Cheung et al 2013 | M |
| Malta | Atlantic bluefin tuna | *Thunnus thynnus* | 228 | 0.82 | 19 | 27 | 24 | Cheung et al 2013 | M |
| Malta | Atlantic bluefin tuna | *Thunnus thynnus* | 227 | 0.82 | 19 | 27 | 24 | Cheung et al 2013 | M |
| Malta | Chub mackerel | *Scomber japonicus* | 168 | 0.31 | 18 | 27 | 23 | Cheung et al 2013 | M |
| Malta | Chub mackerel | *Scomber japonicus* | 168 | 0.31 | 18 | 27 | 23 | Cheung et al 2013 | M |
| Malta | Clupeoids nei | *non specific* | 44 | NA | NA | NA | NA | NA | M |
| Malta | Bogue | *Boops boops* | 35 | 0.41 | 16 | 24 | 20 | Cheung et al 2013 | M |
| Malta | Bogue | *Boops boops* | 35 | 0.41 | 16 | 24 | 20 | Cheung et al 2013 | M |
| Malta | Scorpionfishes nei | *non specific* | 28 | NA | NA | NA | NA | NA | M |
| Malta | Scorpionfishes nei | *non specific* | 28 | NA | NA | NA | NA | NA | M |
| Malta | Octopuses, etc. nei | *non specific* | 27 | NA | NA | NA | NA | NA | M |
| Malta | Octopuses, etc. nei | *non specific* | 27 | NA | NA | NA | NA | NA | M |
| Malta | Surmullet | *European common squid* | 27 | 0.39 | 13 | 23 | 19 | Cheung et al 2013 | M |
| Malta | Surmullet | *Mullus surmuletus* | 27 | 0.39 | 13 | 23 | 19 | Cheung et al 2013 | M |
| Malta | Giant red shrimp | *European common squid* | 23 | 0.10 | NA | NA | 20 | Sealifebase | M |
| Malta | Giant red shrimp | *Aristaeomorpha foliacea* | 23 | 0.10 | NA | NA | 20 | Cheung et al 2013 | M |
| Malta | Marine fishes nei | *non specific* | 21 | NA | NA | NA | NA | NA | M |
| Malta | Frigate and bullet tunas | *Auxis rochei* | 17 | 0.27 | NA | NA | 27 | Fishbase | M |
| Malta | Silver scabbardfish | *Lepidopus caudatus* | 17 | 0.53 | 19 | 27 | 24 | Cheung et al 2013 | M |
| Malta | Common cuttlefish | *Sepia officinalis* | 16 | 0.30 | 15 | 23 | 19 | Cheung et al 2013 | M |
| Malta | Albacore | *Thunnus alalunga* | 15 | 0.58 | 20 | 26 | 23 | Cheung et al 2013 | M |
| Malta | Longnose spurdog | *Squalus blainville* | 15 | 0.67 | 11 | 18 | 13 | Fishbase | M |
| Malta | Deep-water rose shrimp | *Parapenaeus longirostris* | 14 | 0.10 | 23 | 27 | 25 | Cheung et al 2013 | M |
| Malta | Red porgy | *Pagrus pagrus* | 13 | 0.66 | 21 | 27 | 25 | Cheung et al 2013 | M |
| Malta | Natantian decapods nei | *non specific* | 12 | NA | NA | NA | NA | NA | M |
| Malta | European hake | *Merluccius merluccius* | 11 | 0.64 | 15 | 20 | 18 | Cheung et al 2013 | M |
| Malta | Thornback ray | *Raja clavata* | 11 | 0.72 | 14 | 20 | 17 | Cheung et al 2013 | M |
| Malta | Groupers nei | *non specific* | 10 | NA | NA | NA | NA | NA | M |
| Malta | Common pandora | *Pagellus erythrinus* | 10 | 0.40 | 16 | 20 | 18 | Cheung et al 2013 | M |
| Malta | Red mullet | *Mullus barbatus* | 10 | 0.29 | 12 | 22 | 17 | Cheung et al 2013 | M |
| Malta | Surmullets(=Red mullets) nei | *Mullus surmuletus* | 10 | NA | NA | NA | NA | NA | M |
| Malta | European squid | *Loligo vulgaris vulgaris* | 10 | 0.30 | NA | NA | NA | Sealifebase | M |
| Malta | Shads nei | *non specific* | 9 | NA | NA | NA | NA | NA | M |
| Malta | Dogfish sharks nei | *non specific* | 8 | NA | NA | NA | NA | NA | M |
| Malta | Picarels nei | *non specific* | 8 | NA | NA | NA | NA | NA | M |
| Netherlands | Atlantic herring | *Clupea harengus* | 84543 | 0.39 | 8 | 11 | 5 | Cheung et al 2013 | M |
| Netherlands | Atlantic horse mackerel | *Trachurus trachurus* | 64287 | 0.59 | 14 | 22 | 18 | Cheung et al 2013 | M |
| Netherlands | Blue whiting(=Poutassou) | *Micromesistius poutassou* | 59456 | 0.33 | 10 | 19 | 15 | Cheung et al 2013 | M |
| Netherlands | European plaice | *Pleuronectes platessus* | 26535 | 0.71 | 9 | 15 | 12 | Cheung et al 2013 | M |
| Netherlands | Atlantic mackerel | *Scomber scombrus* | 26452 | 0.44 | 8 | 16 | 12 | Cheung et al 2013 | M |
| Netherlands | Common shrimp | *Crangon crangon* | 17759 | 0.10 | 9 | 14 | 11 | Cheung et al 2013 | M |
| Netherlands | Common sole | *Solea solea* | 9683 | 0.36 | 12 | 19 | 16 | Cheung et al 2013 | M |
| Norway | Atlantic herring | *Clupea harengus* | 740654 | 0.39 | 8 | 11 | 5 | Cheung et al 2013 | M |
| Norway | Blue whiting(=Poutassou) | *Micromesistius poutassou* | 404772 | 0.33 | 10 | 19 | 15 | Cheung et al 2013 | M |
| Norway | Atlantic cod | *Gadus morhua* | 297536 | 0.65 | 3 | 9 | 6 | Cheung et al 2013 | M |
| Norway | Saithe(=Pollock) | *Pollachius virens* | 204567 | 0.59 | 4 | 11 | 6 | Cheung et al 2013 | M |
| Norway | Atlantic mackerel | *Scomber scombrus* | 166748 | 0.44 | 8 | 16 | 12 | Cheung et al 2013 | M |
| Norway | Brown seaweeds | *Non specific* | 150653 | NA | NA | NA | NA | Cheung et al 2013 | M |
| Norway | Capelin | *Mallotus villosus* | 143363 | 0.23 | 1 | 5 | 3 | Cheung et al 2013 | M |
| Norway | Haddock | *Melanogrammus aeglefinus* | 99473 | 0.47 | 5 | 10 | 7 | Cheung et al 2013 | M |
| Poland | European sprat | *Sprattus sprattus* | 67605 | 0.25 | 9 | 16 | 12 | Cheung et al 2013 | M |
| Poland | Atlantic herring | *Clupea harengus* | 24554 | 0.39 | 8 | 11 | 5 | Cheung et al 2013 | M |
| Poland | Atlantic cod | *Gadus morhua* | 15248 | 0.65 | 3 | 9 | 6 | Cheung et al 2013 | M |
| Poland | European flounder | *Platichthys flesus* | 10388 | 0.42 | 10 | 15 | 12 | Cheung et al 2013 | M |
| Portugal | European pilchard(=Sardine) | *Sardina pilchardus* | 58617 | 0.27 | 13 | 19 | 16 | Cheung et al 2013 | M |
| Portugal | Chub mackerel | *Scomber japonicus* | 25058 | 0.31 | 18 | 27 | 23 | Cheung et al 2013 | M |
| Portugal | Atlantic horse mackerel | *Trachurus trachurus* | 15361 | 0.59 | 14 | 22 | 18 | Cheung et al 2013 | M |
| Portugal | Common octopus | *Octopus vulgaris* | 7153 | 0.78 | 23 | 26 | 28 | Cheung et al 2013 | M |
| Portugal | Skipjack tuna | *Katsuwonus pelamis* | 4647 | 0.40 | 24 | 27 | 26 | Cheung et al 2013 | M |
| Portugal | Atlantic cod | *Gadus morhua* | 4235 | 0.65 | 3 | 9 | 6 | Cheung et al 2013 | M |
| Portugal | Blue jack mackerel | *Trachurus picturatus* | 3967 | 0.68 | 18 | 24 | 21 | Cheung et al 2013 | M |
| Portugal | Atlantic mackerel | *Scomber scombrus* | 3873 | 0.44 | 8 | 16 | 12 | Cheung et al 2013 | M |
| Portugal | Blue whiting(=Poutassou) | *Micromesistius poutassou* | 3350 | 0.33 | 10 | 19 | 15 | Cheung et al 2013 | M |
| Portugal | Black scabbardfish | *Aphanopus carbo* | 3103 | 0.64 | 7 | 15 | 11 | Cheung et al 2013 | M |
| Portugal | Pouting(=Bib) | *Trisopterus luscus* | 2873 | 0.44 | 10 | 14 | 12 | Cheung et al 2013 | M |
| Portugal | Blue shark | *Prionace glauca* | 2812 | 0.77 | 18 | 26 | 23 | Cheung et al 2013 | M |
| Portugal | Bigeye tuna | *Thunnus obesus* | 2613 | 0.56 | 24 | 27 | 26 | Cheung et al 2013 | M |
| Portugal | Octopuses, etc. nei | *non specific* | 2600 | NA | NA | NA | NA | NA | M |
| Portugal | European hake | *Merluccius merluccius* | 2375 | 0.64 | 15 | 20 | 18 | Cheung et al 2013 | M |
| Portugal | Common edible cockle | *Cerastoderma edule* | 2322 | 0.24 | 8 | 23 | 15 | Newel 1980 | M |
| Portugal | European conger | *Conger conger* | 1688 | 0.86 | 9 | 22 | 16 | Cheung et al 2013 | M |
| Portugal | Common cuttlefish | *Sepia officinalis* | 1546 | 0.30 | 15 | 23 | 19 | Cheung et al 2013 | M |
| Portugal | Atlantic redfishes nei | *Sebastes marinus* | 1441 | 0.56 | 3 | 6 | 5 | Cheung et al 2013 | M |
| Portugal | Blackspot(=red) seabream | *Pagellus bogaraveo* | 1051 | 0.57 | 16 | 20 | 18 | Cheung et al 2013 | M |
| Portugal | Beaked redfish | *Sebastes mentella* | 1042 | 0.56 | 2 | 7 | 4 | Fishbase | M |
| Portugal | Solid surf clam | *Spisula solida* | 1009 | 0.10 | 9 | 12 | 11 | Cheung et al 2013 | M |
| Portugal | Axillary seabream | *Pagellus acarne* | 879 | 0.43 | 18 | 20 | 19 | Cheung et al 2013 | M |
| Portugal | Swordfish | *Xiphias gladius* | 852 | 0.72 | 23 | 27 | 26 | Cheung et al 2013 | M |
| Portugal | Shortfin mako | *Isurus oxyrinchus* | 794 | 0.83 | 22 | 28 | 25 | Cheung et al 2013 | M |
| Portugal | Deep-water rose shrimp | *Parapenaeus longirostris* | 701 | 0.10 | 23 | 27 | 25 | Cheung et al 2013 | M |
| Portugal | European anchovy | *Engraulis encrasicolus* | 658 | 0.24 | 15 | 23 | 21 | Cheung et al 2013 | M |
| Portugal | Mullets nei | *non specific* | 651 | NA | NA | NA | NA | NA | M |
| Portugal | Rays and skates nei | *non specific* | 617 | NA | NA | NA | NA | NA | M |
| Portugal | Thornback ray | *Raja clavata* | 588 | 0.72 | 14 | 20 | 17 | Cheung et al 2013 | M |
| Portugal | Wreckfish | *Polyprion americanus* | 558 | 0.72 | 15 | 21 | 18 | Cheung et al 2013 | M |
| Portugal | Finfishes nei | *non specific* | 553 | NA | NA | NA | NA | NA | M |
| Portugal | Red seaweeds | *non specific* | 552 | NA | NA | NA | NA | NA | M |
| Portugal | Forkbeard | *Phycis phycis* | 535 | 0.45 | 19 | 20 | 19 | Cheung et al 2013 | M |
| Portugal | Frigate and bullet tunas | *Auxis rochei* | 497 | 0.27 | NA | NA | 27 | Fishbase | M |
| Portugal | Haddock | *Melanogrammus aeglefinus* | 489 | 0.47 | 5 | 10 | 7 | Cheung et al 2013 | M |
| Portugal | Veined squid | *Loligo forbesii* | 471 | 0.56 | 21 | 27 | 25 | Cheung et al 2013 | M |
| Romania | European sprat | *Sprattus sprattus* | 389 | 0.25 | 9 | 16 | 12 | Cheung et al 2013 | M |
| Romania | Sea snails | *Non specific* | 371 | NA | NA | NA | NA | NA | M |
| Romania | European anchovy | *Engraulis encrasicolus* | 59 | 0.24 | 15 | 23 | 21 | Cheung et al 2013 | M |
| Romania | Turbot | *Scophthalmus maximus* | 44 | 0.43 | 9 | 14 | 11 | Cheung et al 2013 | M |
| Romania | Whiting | *Merlangius merlangus* | 39 | 0.37 | 9 | 14 | 11 | Cheung et al 2013 | M |
| Romania | Pontic shad | *Alosa pontica* | 29 | 0.35 | 13 | 14 | 13 | Cheung et al 2013 | M |
| Slovenia | European pilchard(=Sardine) | *Sardina pilchardus* | 256 | 0.27 | 13 | 19 | 16 | Cheung et al 2013 | M |
| Slovenia | European anchovy | *Engraulis encrasicolus* | 209 | 0.24 | 15 | 23 | 21 | Cheung et al 2013 | M |
| Slovenia | Whiting | *Merlangius merlangus* | 49 | 0.37 | 9 | 14 | 11 | Cheung et al 2013 | M |
| Slovenia | Horned and musky octopuses | *Eledone cirrhosa* | 19 | 0.30 | 16 | 20 | 18 | Cheung et al 2013 | M |
| Slovenia | Common cuttlefish | *Sepia officinalis* | 17 | 0.30 | 15 | 23 | 19 | Cheung et al 2013 | M |
| Slovenia | European sprat | *Sprattus sprattus* | 16 | 0.25 | 9 | 16 | 12 | Cheung et al 2013 | M |
| Slovenia | Mullets nei | *non specific* | 14 | NA | NA | NA | NA | NA | M |
| Slovenia | Various squids nei | *European common squid* | 12 | 0.10 | NA | NA | NA | Sealifebase | M |
| Slovenia | Common sole | *Solea solea* | 10 | 0.36 | 12 | 19 | 16 | Cheung et al 2013 | M |
| Slovenia | Golden grey mullet | *Liza aurata* | 7 | 0.35 | 13 | 19 | 16 | Cheung et al 2013 | M |
| Slovenia | Gilthead seabream | *Sparus auratus* | 7 | 0.40 | 22 | 28 | 26 | Cheung et al 2013 | M |
| Slovenia | Common pandora | *Pagellus erythrinus* | 6 | 0.40 | 16 | 20 | 18 | Cheung et al 2013 | M |
| Spain | European pilchard(=Sardine) | *Sardina pilchardus* | 54018 | 0.27 | 13 | 19 | 16 | Cheung et al 2013 | M |
| Spain | Jack and horse mackerels nei | *Trachurus mediterraneus* | 36088 | 0.47 | 18 | 20 | 19 | Cheung et al 2013 | M |
| Spain | European hake | *Merluccius merluccius* | 27452 | 0.64 | 15 | 20 | 18 | Cheung et al 2013 | M |
| Spain | Blue whiting(=Poutassou) | *Micromesistius poutassou* | 26470 | 0.33 | 10 | 19 | 15 | Cheung et al 2013 | M |
| Spain | Atlantic mackerel | *Scomber scombrus* | 24394 | 0.44 | 8 | 16 | 12 | Cheung et al 2013 | M |
| Spain | Mackerels nei | *non specific* | 23554 | NA | NA | NA | NA | NA | M |
| Spain | European anchovy | *Engraulis encrasicolus* | 20149 | 0.24 | 15 | 23 | 21 | Cheung et al 2013 | M |
| Spain | Albacore | *Thunnus alalunga* | 13580 | 0.58 | 20 | 26 | 23 | Cheung et al 2013 | M |
| Spain | Pelagic fishes nei | *non specific* | 11571 | NA | NA | NA | NA | NA | M |
| Spain | Atlantic cod | *Gadus morhua* | 11435 | 0.65 | 3 | 9 | 6 | Cheung et al 2013 | M |
| Spain | Groundfishes nei | *non specific* | 9953 | NA | NA | NA | NA | NA | M |
| Spain | Octopuses, etc. nei | *non specific* | 9623 | NA | NA | NA | NA | NA | M |
| Spain | Blue shark | *Prionace glauca* | 9047 | 0.77 | 18 | 26 | 23 | Cheung et al 2013 | M |
| Spain | Marine fishes nei | *non specific* | 8320 | NA | NA | NA | NA | NA | M |
| Spain | Finfishes nei | *non specific* | 7760 | NA | NA | NA | NA | NA | M |
| Spain | Megrims nei | *non specific* | 6137 | NA | NA | NA | NA | NA | M |
| Spain | Monkfishes nei | *non specific* | 6103 | NA | NA | NA | NA | NA | M |
| Spain | Chub mackerel | *Scomber japonicus* | 5679 | 0.31 | 18 | 27 | 23 | Cheung et al 2013 | M |
| Spain | Atlantic pomfret | *Brama brama* | 5644 | 0.71 | 20 | 27 | 24 | Cheung et al 2013 | M |
| Spain | European conger | *Conger conger* | 4905 | 0.86 | 9 | 22 | 16 | Cheung et al 2013 | M |
| Spain | Bogue | *Boops boops* | 4868 | 0.41 | 16 | 24 | 20 | Cheung et al 2013 | M |
| Spain | Sardinellas nei | *non specific* | 4032 | NA | NA | NA | NA | NA | M |
| Spain | Atlantic bluefin tuna | *Thunnus thynnus* | 3874 | 0.82 | 19 | 27 | 24 | Cheung et al 2013 | M |
| Spain | Atlantic horse mackerel | *Trachurus trachurus* | 3803 | 0.59 | 14 | 22 | 18 | Cheung et al 2013 | M |
| Spain | Northern shortfin squid | *Illex illecebrosus* | 3636 | NA | 12 | 21 | 17 | Cheung et al 2013 | M |
| Spain | Roundnose grenadier | *Coryphaenoides rupestris* | 3375 | 0.67 | 6 | 13 | 10 | Cheung et al 2013 | M |
| Spain | Various squids nei | *European common squid* | 3343 | 0.10 | NA | NA | NA | Sealifebase | M |
| Spain | Gadiformes nei | *non specific* | 3317 | NA | NA | NA | NA | NA | M |
| Spain | Swordfish | *Xiphias gladius* | 3229 | 0.72 | 23 | 27 | 26 | Cheung et al 2013 | M |
| Spain | Striped venus | *Venus gallina* | 3219 | 0.10 | 12 | 17 | 14 | Cheung et al 2013 | M |
| Spain | Witch flounder | *Glyptocephalus cynoglossus* | 2884 | 0.68 | 4 | 9 | 7 | Cheung et al 2013 | M |
| Spain | Clams, etc. nei | *non specific* | 2698 | NA | NA | NA | NA | NA | M |
| Spain | Frigate and bullet tunas | *Auxis rochei* | 2694 | 0.27 | NA | NA | 27 | Fishbase | M |
| Spain | Rays and skates nei | *non specific* | 2611 | NA | NA | NA | NA | NA | M |
| Spain | Pouting(=Bib) | *Trisopterus luscus* | 2368 | 0.44 | 10 | 14 | 12 | Cheung et al 2013 | M |
| Spain | Common edible cockle | *Cerastoderma edule* | 2337 | 0.24 | 8 | 23 | 15 | Newel 1980 | M |
| Spain | Beaked redfish | *Sebastes mentella* | 1967 | 0.56 | 2 | 7 | 4 | Fishbase | M |
| Spain | Blackbelly rosefish | *Helicolenus dactylopterus* | 1933 | 0.54 | 18 | 26 | 22 | Cheung et al 2013 | M |
| Spain | Lemon sole | *Microstomus kitt* | 1828 | 0.34 | 3 | 10 | 6 | Cheung et al 2013 | M |
| Spain | Ling | *Molva molva* | 1748 | 0.77 | 10 | 17 | 14 | Cheung et al 2013 | M |
| Spain | John dory | *Zeus faber* | 1662 | 0.68 | 19 | 26 | 23 | Cheung et al 2013 | M |
| Spain | Forkbeard | *Phycis phycis* | 1645 | 0.45 | 19 | 20 | 19 | Cheung et al 2013 | M |
| Spain | Common squids nei | *Loligo vulgaris* | 1608 | 0.19 | 17 | 19 | 18 | Cheung et al 2013 | M |
| Spain | Surmullets(=Red mullets) nei | *non specific* | 1607 | NA | NA | NA | NA | NA | M |
| Spain | Common cuttlefish | *Sepia officinalis* | 1509 | 0.30 | 15 | 23 | 19 | Cheung et al 2013 | M |
| Spain | Atlantic redfishes nei | *Sebastes marinus* | 1481 | 0.56 | 3 | 6 | 5 | Cheung et al 2013 | M |
| Spain | Baird's slickhead | *Alepocephalus bairdii* | 1441 | 0.70 | 7 | 17 | 12 | Cheung et al 2013 | M |
| Spain | Gurnards, searobins nei | *non specific* | 1369 | NA | NA | NA | NA | NA | M |
| Spain | Deep-water rose shrimp | *Parapenaeus longirostris* | 1308 | 0.10 | 23 | 27 | 25 | Cheung et al 2013 | M |
| Spain | Atlantic saury | *Scomberesox saurus* | 1244 | 0.25 | 14 | 24 | 20 | Cheung et al 2013 | M |
| Spain | Greater forkbeard | *Phycis blennoides* | 1202 | 0.63 | 12 | 19 | 16 | Cheung et al 2013 | M |
| Spain | Norway lobster | *Nephrops norvegicus* | 1166 | 0.14 | 14 | 20 | 17 | Cheung et al 2013 | M |
| Sweden | Atlantic herring | *Clupea harengus* | 84126 | 0.39 | 8 | 11 | 5 | Cheung et al 2013 | M |
| Sweden | European sprat | *Sprattus sprattus* | 79901 | 0.25 | 9 | 16 | 12 | Cheung et al 2013 | M |
| Sweden | Sandeels(=Sandlances) nei | *Ammodytes tobianus* | 20554 | 0.23 | 11 | 16 | 13 | Cheung et al 2013 | M |
| Sweden | Atlantic cod | *Gadus morhua* | 12270 | 0.65 | 3 | 9 | 6 | Cheung et al 2013 | M |
| Turkey | European anchovy | *Engraulis encrasicolus* | 226136 | 0.24 | 15 | 23 | 21 | Cheung et al 2013 | M |
| Turkey | Striped venus | *Venus gallina* | 32088 | 0.10 | 12 | 17 | 14 | Cheung et al 2013 | M |
| Turkey | European sprat | *Sprattus sprattus* | 30045 | 0.25 | 9 | 16 | 12 | Cheung et al 2013 | M |
| Turkey | European pilchard(=Sardine) | *Sardina pilchardus* | 22753 | 0.27 | 13 | 19 | 16 | Cheung et al 2013 | M |
| Turkey | Atlantic bonito | *Sarda sarda* | 19365 | 0.33 | 18 | 26 | 23 | Cheung et al 2013 | M |
| Turkey | Mediterranean horse mackerel | *Trachurus mediterraneus* | 18396 | 0.47 | 18 | 20 | 19 | Cheung et al 2013 | M |
| Turkey | Marine molluscs nei | *non specific* | 10310 | NA | NA | NA | NA | NA | M |
| Turkey | Whiting | *Merlangius merlangus* | 10116 | 0.37 | 9 | 14 | 11 | Cheung et al 2013 | M |
| Turkey | Bluefish | *Pomatomus saltatrix/saltator* | 8403 | 0.63 | 25 | 28 | 27 | Cheung et al 2013 | M |
| Turkey | Atlantic horse mackerel | *Trachurus trachurus* | 8374 | 0.59 | 14 | 22 | 18 | Cheung et al 2013 | M |
| Turkey | Mullets nei | *non specific* | 5490 | NA | NA | NA | NA | NA | M |
| United Kingdom | Atlantic mackerel | *Scomber scombrus* | 163413 | 0.44 | 8 | 16 | 12 | Cheung et al 2013 | M |
| United Kingdom | Atlantic herring | *Clupea harengus* | 88384 | 0.39 | 8 | 11 | 5 | Cheung et al 2013 | M |
| United Kingdom | Blue whiting(=Poutassou) | *Micromesistius poutassou* | 38992 | 0.33 | 10 | 19 | 15 | Cheung et al 2013 | M |
| United Kingdom | Haddock | *Melanogrammus aeglefinus* | 35913 | 0.47 | 5 | 10 | 7 | Cheung et al 2013 | M |
| United Kingdom | Norway lobster | *Nephrops norvegicus* | 35898 | 0.14 | 14 | 20 | 17 | Cheung et al 2013 | M |
| United Kingdom | Great Atlantic scallop | *Pecten maximus* | 27204 | 0.26 | 11 | 15 | 12 | Cheung et al 2013 | M |
| United Kingdom | Edible crab | *Cancer pagurus* | 26267 | 0.10 | 11 | 16 | 14 | Cheung et al 2013 | M |
| United Kingdom | Atlantic cod | *Gadus morhua* | 23064 | 0.65 | 3 | 9 | 6 | Cheung et al 2013 | M |
| United Kingdom | European plaice | *Pleuronectes platessus* | 16174 | 0.71 | 9 | 15 | 12 | Cheung et al 2013 | M |
| United Kingdom | Whelk | *Buccinum undatum* | 14819 | NA | 10 | 12 | 11 | Cheung et al 2013 | M |
| United Kingdom | Angler(=Monk) | *Lophius piscatorius* | 14230 | 0.72 | 10 | 17 | 14 | Cheung et al 2013 | M |
| United Kingdom | Saithe(=Pollock) | *Pollachius virens* | 14200 | 0.59 | 4 | 11 | 6 | Cheung et al 2013 | M |
| United Kingdom | Atlantic horse mackerel | *Trachurus trachurus* | 13725 | 0.59 | 14 | 22 | 18 | Cheung et al 2013 | M |
| United Kingdom | Queen scallop | *Chlamys opercularis* | 12316 | 0.22 | 11 | 13 | 12 | Cheung et al 2013 | M |
| United Kingdom | Whiting | *Merlangius merlangus* | 10579 | 0.37 | 9 | 14 | 11 | Cheung et al 2013 | M |
| United Kingdom | Common edible cockle | *Cerastoderma edule* | 8395 | 0.24 | 8 | 23 | 15 | Newel 1980 | M |
| United Kingdom | Blue mussel | *Mytilus edulis* | 6903 | 0.36 | 4 | 11 | 8 | Cheung et al 2013 | M |
| United Kingdom | European hake | *Merluccius merluccius* | 6717 | 0.64 | 15 | 20 | 18 | Cheung et al 2013 | M |
| United Kingdom | European sprat | *Sprattus sprattus* | 4878 | 0.25 | 9 | 16 | 12 | Cheung et al 2013 | M |
| Bulgaria | Mediterranean mussel | *Mytilus galloprovincialis* | 797 | 0.10 | 14 | 19 | 15 | Cheung et al 2013 | A |
| Croatia | European seabass | *Dicentrarchus labrax* | 2643 | 0.49 | 10 | 17 | 14 | Cheung et al 2013 | A |
| Croatia | Mediterranean mussel | *Mytilus galloprovincialis* | 2091 | 0.10 | 14 | 19 | 15 | Cheung et al 2013 | A |
| Croatia | Gilthead seabream | *Sparus auratus* | 1893 | 0.40 | 22 | 28 | 26 | Cheung et al 2013 | A |
| Cyprus | Gilthead seabream | *Sparus auratus* | 2390 | 0.40 | 22 | 28 | 26 | Cheung et al 2013 | A |
| Cyprus | European seabass | *Dicentrarchus labrax* | 1009 | 0.49 | 10 | 17 | 14 | Cheung et al 2013 | A |
| Denmark | Rainbow trout | *Oncorhynchus mykiss* | 10190 | 0.36 | 6 | 12 | 9 | Cheung et al 2013 | A |
| Denmark | Blue mussel | *Mytilus edulis* | 941 | 0.36 | 4 | 11 | 8 | Cheung et al 2013 | A |
| Denmark | Brown seaweeds | *non specific* | 627 | NA | NA | NA | NA | NA | A |
| Estonia | Rainbow trout | *Oncorhynchus mykiss* | 0 | 0.36 | 6 | 12 | 9 | Cheung et al 2013 | A |
| Faroe Islands | Atlantic salmon | *Salmo salar* | 48174 | 0.62 | 6 | 12 | 9 | Cheung et al 2013 | A |
| Finland | Rainbow trout | *Oncorhynchus mykiss* | 10171 | 0.36 | 6 | 12 | 9 | Cheung et al 2013 | A |
| France | Pacific cupped oyster | *Crassostrea gigas* | 97753 | 0.35 | 10 | 16 | 13 | Cheung et al 2013 | A |
| France | Blue mussel | *Mytilus edulis* | 59799 | 0.36 | 4 | 11 | 8 | Cheung et al 2013 | A |
| France | Mediterranean mussel | *Mytilus galloprovincialis* | 14337 | 0.10 | 14 | 19 | 15 | Cheung et al 2013 | A |
| Germany | Blue mussel | *Mytilus edulis* | 8149 | 0.36 | 4 | 11 | 8 | Cheung et al 2013 | A |
| Greece | Gilthead seabream | *Sparus auratus* | 50547 | 0.40 | 22 | 28 | 26 | Cheung et al 2013 | A |
| Greece | European seabass | *Dicentrarchus labrax* | 34003 | 0.49 | 10 | 17 | 14 | Cheung et al 2013 | A |
| Greece | Mediterranean mussel | *Mytilus galloprovincialis* | 21359 | 0.10 | 14 | 19 | 15 | Cheung et al 2013 | A |
| Iceland | Atlantic salmon | *Salmo salar* | 2966 | 0.62 | 6 | 12 | 9 | Cheung et al 2013 | A |
| Iceland | Arctic char | *Salvelinus alpinus alpinus* | 2209 | 0.74 | 0 | 3 | 10 | Cheung et al 2013 | A |
| Iceland | Atlantic cod | *Gadus morhua* | 1048 | 0.65 | 3 | 9 | 6 | Cheung et al 2013 | A |
| Ireland | Blue mussel | *Mytilus edulis* | 26086 | 0.36 | 4 | 11 | 8 | Cheung et al 2013 | A |
| Ireland | Atlantic salmon | *Salmo salar* | 11743 | 0.62 | 6 | 12 | 9 | Cheung et al 2013 | A |
| Ireland | Pacific cupped oyster | *Crassostrea gigas* | 6977 | 0.35 | 10 | 16 | 13 | Cheung et al 2013 | A |
| Italy | Mediterranean mussel | *Mytilus galloprovincialis* | 68308 | 0.10 | 14 | 19 | 15 | Cheung et al 2013 | A |
| Italy | Japanese carpet shell | *Ruditapes philippinarum* | 39004 | 0.10 | 16 | 20 | 18 | Cheung et al 2013 | A |
| Italy | European seabass | *Dicentrarchus labrax* | 6972 | 0.49 | 10 | 17 | 14 | Cheung et al 2013 | A |
| Malta | Gilthead seabream | *Sparus auratus* | 1595 | 0.40 | 22 | 28 | 26 | Cheung et al 2013 | A |
| Malta | Atlantic bluefin tuna | *Thunnus thynnus* | 934 | 0.82 | 19 | 27 | 24 | Cheung et al 2013 | A |
| Malta | Marine fishes nei | *non specific* | 206 | NA | NA | NA | NA | NA | A |
| Malta | European seabass | *Dicentrarchus labrax* | 118 | 0.49 | 10 | 17 | 14 | Cheung et al 2013 | A |
| Malta | European seabass | *Dicentrarchus labrax* | 118 | 0.49 | 10 | 17 | 14 | Cheung et al 2013 | A |
| Malta | Turbot | *Scophthalmus maximus* | 0 | 0.43 | 9 | 14 | 11 | Cheung et al 2013 | A |
| Netherlands | Blue mussel | *Mytilus edulis* | 47723 | 0.36 | 4 | 11 | 8 | Cheung et al 2013 | A |
| Norway | Atlantic salmon | *Salmo salar* | 889838 | 0.62 | 6 | 12 | 9 | Cheung et al 2013 | A |
| Portugal | Grooved carpet shell | *Ruditapes decussatus* | 2213 | 0.30 | 13 | 22 | 17 | Cheung et al 2013 | A |
| Portugal | Turbot | *Scophthalmus maximus* | 1675 | 0.43 | 9 | 14 | 11 | Cheung et al 2013 | A |
| Portugal | Gilthead seabream | *Sparus auratus* | 1413 | 0.40 | 22 | 28 | 26 | Cheung et al 2013 | A |
| Portugal | European seabass | *Dicentrarchus labrax* | 859 | 0.49 | 10 | 17 | 14 | Cheung et al 2013 | A |
| Portugal | Mediterranean mussel | *Mytilus galloprovincialis* | 462 | 0.1 | 14 | 19 | 15 | Cheung et al 2013 | A |
| Portugal | Pacific cupped oyster | *Crassostrea gigas* | 398 | 0.35 | 10 | 16 | 13 | Cheung et al 2013 | A |
| Romania | Turbot | *Engraulis encrasicolus* | 3 | 0.43 | 9 | 14 | 11 | Cheung et al 2013 | A |
| Romania | Mediterranean mussel | *Mytilus galloprovincialis* | 1 | 0.10 | 14 | 19 | 15 | Cheung et al 2013 | A |
| Slovenia | Mediterranean mussel | *Mytilus galloprovincialis* | 267 | 0.10 | 14 | 19 | 15 | Cheung et al 2013 | A |
| Slovenia | European seabass | *Dicentrarchus labrax* | 49 | 0.49 | 10 | 17 | 14 | Cheung et al 2013 | A |
| Spain | Mediterranean mussel | *Mytilus galloprovincialis* | 199169 | 0.1 | 14 | 19 | 15 | Cheung et al 2013 | A |
| Spain | Gilthead seabream | *Sparus auratus* | 16546 | 0.40 | 22 | 28 | 26 | Cheung et al 2013 | A |
| Sweden | Rainbow trout | *Oncorhynchus mykiss* | 2188 | 0.36 | 6 | 12 | 9 | Cheung et al 2013 | A |
| Sweden | Blue mussel | *Mytilus edulis* | 1555 | 0.36 | 4 | 11 | 8 | Cheung et al 2013 | A |
| Turkey | European seabass | *Dicentrarchus labrax* | 49619 | 0.49 | 10 | 17 | 14 | Cheung et al 2013 | A |
| Turkey | Gilthead seabream | *Sparus auratus* | 30857 | 0.40 | 22 | 28 | 26 | Cheung et al 2013 | A |
| United Kingdom | Atlantic salmon | *Salmo salar* | 147944 | 0.62 | 6 | 12 | 9 | Cheung et al 2013 | A |
| United Kingdom | Blue mussel | *Mytilus edulis* | 27024 | 0.36 | 4 | 11 | 8 | Cheung et al 2013 | A |
| Austria | Rainbow trout | *Oncorhynchus mykiss* | 1413 | 0.36 | 6 | 12 | 9 | Cheung et al 2013 | F |
| Austria | Common carp | *Cyprinus carpio* | 435 | 0.46 | 15 | 32 | 18 | Tank et al. 2000 | F |
| Austria | Freshwater fishes nei | *non specific* | 353 | NA | NA | NA | NA | NA | F |
| Austria | Brook trout | *Salvelinus fontinalis* | 314 | 0.43 | NA | NA | 13 | Cheung et al 2013 | F |
| Austria | Sea trout | *Salmo trutta* | 148 | 0.60 | 9 | 10 | 9 | Cheung et al 2013 | F |
| Austria | North African catfish | *Clarias gariepinus* | 98 | 0.79 | 8 | 35 | 21 | Fishbase | F |
| Belgium | Roach | *Rutilus rutilus* | 111 | 0.53 | 10 | 20 | 15 | Fishbase | F |
| Belgium | Freshwater fishes nei | *non specific* | 93 | NA | NA | NA | NA | NA | F |
| Belgium | Sea trout | *Salmo trutta* | 84 | 0.60 | 9 | 10 | 9 | Cheung et al 2013 | F |
| Belgium | Rainbow trout | *Oncorhynchus mykiss* | 79 | 0.36 | 6 | 12 | 9 | Cheung et al 2013 | F |
| Belgium | Common carp | *Cyprinus carpio* | 78 | 0.46 | 15 | 32 | 18 | Tank et al. 2000 | F |
| Belgium | Aquatic invertebrates nei | *non specific* | 53 | NA | NA | NA | NA | NA | F |
| Belgium | Freshwater bream | *Abramis Brama* | 44 | 0.62 | 10 | 24 | 16 | Fishbase | F |
| Belgium | Pike-perch | *Sander lucioperca* | 40 | 0.62 | 6 | 22 | 14 | Fishbase | F |
| Belgium | Cyprinids nei | *non specific* | 36 | NA | NA | NA | NA | NA | F |
| Belgium | European eel | *Anguilla anguilla* | 30 | 0.64 | 4 | 20 | 12 | Fishbase | F |
| Belgium | European perch | *Perca fluviatilis* | 20 | 0.50 | 10 | 22 | 16 | Fishbase | F |
| Bulgaria | Common carp | *Cyprinus carpio* | 2150 | 0.46 | 15 | 32 | 18 | Tank et al. 2000 | F |
| Bulgaria | Rainbow trout | *Oncorhynchus mykiss* | 2064 | 0.36 | 6 | 12 | 9 | Cheung et al 2013 | F |
| Bulgaria | Bighead carp | *Hypophthalmichthys nobilis* | 1180 | 0.66 | 4 | 26 | 15 | Fishbase | F |
| Bulgaria | Goldfish | *Carassius auratus* | 240 | 0.24 | 10 | 30 | 20 | Kneprath and Meade 1968 | F |
| Bulgaria | Danube sturgeon(=Osetr) | *Acipenser gueldenstaedtii* | 201 | 0.87 | 10 | 20 | 11 | Fishbase | F |
| Bulgaria | Grass carp(=White amur) | *Ctenopharyngodon idella* | 150 | 0.65 | NA | 35 | NA | Fishbase | F |
| Bulgaria | Silver carp | *Hypophthalmichthys molitrix* | 79 | 0.55 | 6 | 22 | 15 | Cheung et al 2013 | F |
| Croatia | Common carp | *Cyprinus carpio* | 2068 | 0.46 | 15 | 32 | 18 | Tank et al. 2000 | F |
| Croatia | Rainbow trout | *Oncorhynchus mykiss* | 1546 | 0.36 | 6 | 12 | 9 | Cheung et al 2013 | F |
| Croatia | Bighead carp | *Hypophthalmichthys nobilis* | 253 | 0.66 | 4 | 26 | 15 | Fishbase | F |
| Croatia | Grass carp(=White amur) | *Ctenopharyngodon idella* | 246 | 0.65 | NA | 35 | NA | Fishbase | F |
| Croatia | Silver carp | *Hypophthalmichthys molitrix* | 225 | 0.55 | 6 | 22 | 15 | Cheung et al 2013 | F |
| Czech Republic | Common carp | *Cyprinus carpio* | 20865 | 0.46 | 15 | 32 | 18 | Tank et al. 2000 | F |
| Czech Republic | Rainbow trout | *Oncorhynchus mykiss* | 527 | 0.36 | 6 | 12 | 9 | Cheung et al 2013 | F |
| Czech Republic | Freshwater fishes nei | *non specific* | 510 | NA | NA | NA | NA | NA | F |
| Czech Republic | Grass carp(=White amur) | *Ctenopharyngodon idella* | 381 | 0.65 | NA | 35 | NA | Fishbase | F |
| Denmark | Rainbow trout | *Oncorhynchus mykiss* | 23108 | 0.36 | 6 | 12 | 9 | Cheung et al 2013 | F |
| Estonia | Pike-perch | *Sander lucioperca* | 761 | 0.62 | 6 | 22 | 14 | Fishbase | F |
| Estonia | European perch | *Perca fluviatilis* | 744 | 0.50 | 10 | 22 | 16 | Fishbase | F |
| Estonia | Freshwater bream | *Abramis Brama* | 619 | 0.62 | 10 | 24 | 16 | Fishbase | F |
| Estonia | Rainbow trout | *Oncorhynchus mykiss* | 481 | 0.36 | 6 | 12 | 9 | Cheung et al 2013 | F |
| Estonia | Roach | *Rutilus rutilus* | 268 | 0.53 | 10 | 20 | 15 | Fishbase | F |
| Estonia | Northern pike | *Esox lucius* | 157 | 0.85 | 10 | 28 | 19 | Fishbase | F |
| Estonia | River lamprey | *Lampetra fluviatilis* | 49 | 0.62 | 5 | 18 | 11 | Fishbase | F |
| Faroe Islands | Freshwater fishes nei | *non specific* | 0 | NA | NA | NA | NA | NA | F |
| Finland | European perch | *Perca fluviatilis* | 7582 | 0.50 | 10 | 22 | 16 | Fishbase | F |
| Finland | Northern pike | *Esox lucius* | 6723 | 0.85 | 10 | 28 | 19 | Fishbase | F |
| Finland | Vendace | *Coregonus albula* | 4203 | 0.27 | 8 | 10 | 9 | Cheung et al 2013 | F |
| Finland | Roach | *Rutilus rutilus* | 3345 | 0.53 | 10 | 20 | 15 | Fishbase | F |
| Finland | Pike-perch | *Sander lucioperca* | 2433 | 0.62 | 6 | 22 | 14 | Fishbase | F |
| Finland | Rainbow trout | *Oncorhynchus mykiss* | 1858 | 0.36 | 6 | 12 | 9 | Cheung et al 2013 | F |
| Finland | Freshwater bream | *Abramis Brama* | 1564 | 0.62 | 10 | 24 | 16 | Fishbase | F |
| Finland | European whitefish | *Coregonus lavaretus* | 1208 | 0.51 | 8 | 12 | 10 | Cheung et al 2013 | F |
| Finland | Burbot | *Lota lota* | 680 | 0.66 | 4 | 18 | 11 | Fishbase | F |
| France | Rainbow trout | *Oncorhynchus mykiss* | 32244 | 0.36 | 6 | 12 | 9 | Cheung et al 2013 | F |
| France | Common carp | *Cyprinus carpio* | 4173 | 0.46 | 15 | 32 | 18 | Tank et al. 2000 | F |
| France | Roach | *Rutilus rutilus* | 1900 | 0.53 | 10 | 20 | 15 | Fishbase | F |
| France | Freshwater fishes nei | *non specific* | 1174 | NA | NA | NA | NA | NA | F |
| France | Sea trout | *Salmo trutta* | 910 | 0.60 | 9 | 10 | 9 | Cheung et al 2013 | F |
| Germany | Rainbow trout | *Oncorhynchus mykiss* | 16692 | 0.36 | 6 | 12 | 9 | Cheung et al 2013 | F |
| Germany | Freshwater fishes nei | *non specific* | 15584 | NA | NA | NA | NA | NA | F |
| Germany | Common carp | *Cyprinus carpio* | 9076 | 0.46 | 15 | 32 | 18 | Tank et al. 2000 | F |
| Germany | Cyprinids nei | *non specific* | 1180 | NA | NA | NA | NA | NA | F |
| Greece | Rainbow trout | *Oncorhynchus mykiss* | 2432 | 0.36 | 6 | 12 | 9 | Cheung et al 2013 | F |
| Greece | European eel | *Anguilla anguilla* | 306 | 0.64 | 4 | 20 | 12 | Fishbase | F |
| Greece | Goldfish | *Carassius auratus* | 278 | 0.24 | 10 | 30 | 20 | Kneprath and Meade 1968 | F |
| Greece | Common carp | *Cyprinus carpio* | 230 | 0.46 | 15 | 32 | 18 | Tank et al. 2000 | F |
| Greece | Freshwater fishes nei | *non specific* | 215 | NA | NA | NA | NA | NA | F |
| Greece | Big-scale sand smelt | *Atherina boyeri* | 155 | 0.44 | 11 | 17 | 14 | Cheung et al 2013 | F |
| Greece | Roaches nei | *Non specific* | 136 | NA | NA | NA | NA | NA | F |
| Hungary | Common carp | *Cyprinus carpio* | 13629 | 0.46 | 15 | 32 | 18 | Tank et al. 2000 | F |
| Hungary | North African catfish | *Clarias gariepinus* | 1786 | 0.79 | 8 | 35 | 21 | Fishbase | F |
| Hungary | Silver carp | *Hypophthalmichthys molitrix* | 1526 | 0.55 | 6 | 22 | 15 | Cheung et al 2013 | F |
| Hungary | Cyprinids nei | *non specific* | 1378 | NA | NA | NA | NA | NA | F |
| Hungary | Freshwater fishes nei | *non specific* | 633 | NA | NA | NA | NA | NA | F |
| Hungary | Grass carp(=White amur) | *Ctenopharyngodon idella* | 532 | 0.65 | NA | 35 | NA | Fishbase | F |
| Hungary | Silver carp | *Hypophthalmichthys molitrix* | 519 | 0.55 | 6 | 22 | 15 | Cheung et al 2013 | F |
| Iceland | Arctic char | *Salvelinus alpinus alpinus* | 175 | 0.74 | 0 | 3 | 10 | Cheung et al 2013 | F |
| Iceland | Atlantic salmon | *Salmo salar* | 146 | 0.62 | 6 | 12 | 9 | Cheung et al 2013 | F |
| Iceland | Rainbow trout | *Oncorhynchus mykiss* | 144 | 0.36 | 6 | 12 | 9 | Cheung et al 2013 | F |
| Iceland | Sea trout | *Salmo trutta* | 39 | 0.60 | 9 | 10 | 9 | Cheung et al 2013 | F |
| Ireland | Rainbow trout | *Oncorhynchus mykiss* | 783 | 0.36 | 6 | 12 | 9 | Cheung et al 2013 | F |
| Ireland | Atlantic salmon | *Salmo salar* | 168 | 0.62 | 6 | 12 | 9 | Cheung et al 2013 | F |
| Ireland | European eel | *Anguilla anguilla* | 48 | 0.64 | 4 | 20 | 12 | Fishbase | F |
| Italy | Rainbow trout | *Oncorhynchus mykiss* | 33631 | 0.36 | 6 | 12 | 9 | Cheung et al 2013 | F |
| Italy | Freshwater fishes nei | *non specific* | 2794 | NA | NA | NA | NA | NA | F |
| Italy | Sea trout | *Salmo trutta* | 916 | 0.60 | 9 | 10 | 9 | Cheung et al 2013 | F |
| Italy | Sturgeons nei | *Acipenser sturio* | 835 | 0.86 | NA | NA | NA | Fishbase | F |
| Latvia | Common carp | *Cyprinus carpio* | 488 | 0.46 | 15 | 32 | 18 | Tank et al. 2000 | F |
| Latvia | River lamprey | *Lampetra fluviatilis* | 89 | 0.62 | 5 | 18 | 11 | Fishbase | F |
| Latvia | Freshwater bream | *Abramis Brama* | 64 | 0.62 | 10 | 24 | 16 | Fishbase | F |
| Latvia | Northern pike | *Esox lucius* | 41 | 0.85 | 10 | 28 | 19 | Fishbase | F |
| Latvia | Tench | *Tinca tinca* | 36 | 0.65 | 4 | 24 | 14 | Fishbase | F |
| Latvia | Pike-perch | *Sander lucioperca* | 28 | 0.62 | 6 | 22 | 14 | Fishbase | F |
| Latvia | Sturgeons nei | *Acipenser sturio* | 20 | 0.86 | NA | NA | NA | Fishbase | F |
| Latvia | Roach | *Rutilus rutilus* | 17 | 0.53 | 10 | 20 | 15 | Fishbase | F |
| Latvia | European perch | *Perca fluviatilis* | 14 | 0.50 | 10 | 22 | 16 | Fishbase | F |
| Latvia | Crucian carp | *Carassius carassius* | 14 | 0.38 | 2 | 22 | 13 | Fishbase | F |
| Latvia | Freshwater fishes nei | *Non specific* | 14 | NA | NA | NA | NA | NA | F |
| Lithuania | Common carp | *Cyprinus carpio* | 2930 | 0.46 | 15 | 32 | 18 | Fishbase | F |
| Lithuania | Roach | *Rutilus rutilus* | 432 | 0.53 | 10 | 20 | 15 | Fishbase | F |
| Lithuania | Freshwater bream | *Abramis Brama* | 423 | 0.62 | 10 | 24 | 16 | Fishbase | F |
| Lithuania | European smelt | *Osmerus eperlanus* | 206 | 0.43 | 9 | 11 | 10 | Cheung et al 2013 | F |
| Lithuania | Pike-perch | *Sander lucioperca* | 96 | 0.62 | 6 | 22 | 14 | Cheung et al 2013 | F |
| Lithuania | Vimba bream | *Vimba vimba* | 88 | 0.37 | 10 | 20 | 15 | Fishbase | F |
| Lithuania | European perch | *Perca fluviatilis* | 60 | 0.50 | 10 | 22 | 16 | Fishbase | F |
| Malta | Freshwater fishes nei | *non specific* | 0 | NA | NA | NA | NA | NA | F |
| Netherlands | European eel | *Anguilla anguilla* | 3432 | 0.64 | 4 | 20 | 12 | Fishbase | F |
| Netherlands | North African catfish | *Clarias gariepinus* | 3061 | 0.79 | 8 | 35 | 21 | Fishbase | F |
| Netherlands | European smelt | *Osmerus eperlanus* | 818 | 0.43 | 9 | 11 | 10 | Cheung et al 2013 | F |
| Netherlands | Nile tilapia | *Oreochromis niloticus* | 386 | 0.30 | 14 | 33 | 24 | Fishbase | F |
| Netherlands | European eel | *Anguilla anguilla* | 300 | 0.64 | 4 | 20 | 12 | Fishbase | F |
| Netherlands | Pike-perch | *Sander lucioperca* | 294 | 0.62 | 6 | 22 | 14 | Fishbase | F |
| Norway | Atlantic salmon | *Salmo salar* | 362 | 0.62 | 6 | 12 | 9 | Cheung et al 2013 | F |
| Norway | Sea trout | *Salmo trutta* | 76 | 0.60 | 9 | 10 | 9 | Cheung et al 2013 | F |
| Poland | Common carp | *Cyprinus carpio* | 17314 | 0.46 | 15 | 32 | 18 | Tank et al. 2000 | F |
| Poland | Freshwater fishes nei | *non specific* | 16026 | NA | NA | NA | NA | NA | F |
| Poland | Rainbow trout | *Oncorhynchus mykiss* | 14330 | 0.36 | 6 | 12 | 9 | Cheung et al 2013 | F |
| Poland | Freshwater bream | *Abramis Brama* | 1057 | 0.62 | 10 | 24 | 16 | Fishbase | F |
| Portugal | Rainbow trout | *Oncorhynchus mykiss* | 737 | 0.36 | 6 | 12 | 9 | Cheung et al 2013 | F |
| Romania | Common carp | *Cyprinus carpio* | 3138 | 0.46 | 15 | 32 | 18 | Tank et al. 2000 | F |
| Romania | Goldfish | *Carassius auratus* | 2782 | 0.24 | 10 | 30 | 20 | Kneprath and Meade 1968 | F |
| Romania | Silver carp | *Hypophthalmichthys molitrix* | 1855 | 0.55 | 6 | 22 | 15 | Cheung et al 2013 | F |
| Romania | Bighead carp | *Hypophthalmichthys nobilis* | 1384 | 0.66 | 4 | 26 | 15 | Fishbase | F |
| Romania | Rainbow trout | *Oncorhynchus mykiss* | 1166 | 0.36 | 6 | 12 | 9 | Cheung et al 2013 | F |
| Romania | Freshwater bream | *Abramis Brama* | 605 | 0.62 | 10 | 24 | 16 | Fishbase | F |
| Romania | Pontic shad | *Alosa pontica* | 371 | 0.35 | 13 | 14 | 13 | Cheung et al 2013 | F |
| Romania | Sea trout | *Salmo trutta* | 303 | 0.60 | 9 | 10 | 9 | Cheung et al 2013 | F |
| Romania | Roaches nei | *Non specific* | 218 | NA | NA | NA | NA | NA | F |
| Romania | Common carp | *Cyprinus carpio* | 170 | 0.46 | 15 | 32 | 18 | Tank et al. 2000 | F |
| Romania | Cyprinids nei | *non specific* | 164 | NA | NA | NA | NA | NA | F |
| Romania | Wels(=Som) catfish | *Silurus glanis* | 153 | 0.84 | 4 | 20 | 12 | Fishbase | F |
| Slovakia | Common carp | *Cyprinus carpio* | 1527 | 0.46 | 15 | 32 | 18 | Tank et al. 2000 | F |
| Slovakia | Rainbow trout | *Oncorhynchus mykiss* | 726 | 0.36 | 6 | 12 | 9 | Cheung et al 2013 | F |
| Slovakia | Freshwater breams nei | *Taken values from Freshwater bream* | 81 | 0.62 | 10 | 24 | 16 | Fishbase | F |
| Slovakia | Goldfish | *Carassius auratus* | 65 | 0.24 | 10 | 30 | 20 | Kneprath and Meade 1968 | F |
| Slovakia | Pike-perch | *Sander lucioperca* | 64 | 0.62 | 6 | 22 | 14 | Fishbase | F |
| Slovakia | Northern pike | *Esox lucius* | 57 | 0.85 | 10 | 28 | 19 | Cheung et al 2013 | F |
| Slovakia | Grass carp(=White amur) | *Ctenopharyngodon idella* | 47 | 0.65 | NA | 35 | NA | Fishbase | F |
| Slovenia | Common carp | *Cyprinus carpio* | 2253 | 0.46 | 15 | 32 | 18 | Tank et al. 2000 | F |
| Slovenia | Rainbow trout | *Oncorhynchus mykiss* | 708 | 0.36 | 6 | 12 | 9 | Cheung et al 2013 | F |
| Slovenia | Salmonids nei | *non specific* | 37 | NA | NA | NA | NA | NA | F |
| Slovenia | Rainbow trout | *Oncorhynchus mykiss* | 21 | 0.36 | 6 | 12 | 9 | Cheung et al 2013 | F |
| Slovenia | Silver carp | *Hypophthalmichthys molitrix* | 13 | 0.55 | 6 | 22 | 15 | Cheung et al 2013 | F |
| Slovenia | Brook trout | *Salvelinus fontinalis* | 12 | 0.43 | NA | NA | 13 | Cunjak and Green 1986 | F |
| Slovenia | Cyprinids nei | *non specific* | 11 | NA | NA | NA | NA | NA | F |
| Spain | Rainbow trout | *Oncorhynchus mykiss* | 20612 | 0.36 | 6 | 12 | 9 | Cheung et al 2013 | F |
| Spain | Freshwater fishes nei | *non specific* | 2650 | NA | NA | NA | NA | NA | F |
| Spain | Sea trout | *Salmo trutta* | 1800 | 0.60 | 9 | 10 | 9 | Cheung et al 2013 | F |
| Sweden | Rainbow trout | *Oncorhynchus mykiss* | 4796 | 0.36 | 6 | 12 | 9 | Cheung et al 2013 | F |
| Sweden | Chars nei | *Salvelinus alpinus alpinus* | 879 | 0.74 | 0 | 3 | 10 | Cheung et al 2013 | F |
| Sweden | European perch | *Perca fluviatilis* | 681 | 0.50 | 10 | 22 | 16 | Fishbase | F |
| Sweden | Pike-perch | *Sander lucioperca* | 578 | 0.62 | 6 | 22 | 14 | Fishbase | F |
| Sweden | Northern pike | *Esox lucius* | 483 | 0.85 | 10 | 28 | 19 | Fishbase | F |
| Sweden | Vendace | *Coregonus albula* | 271 | 0.27 | 8 | 10 | 9 | Cheung et al 2013 | F |
| Sweden | Euro-American crayfishes nei | *Homarus gammarus* | 260 | 0.46 | NA | NA | 6 | Sealifebase | F |
| Sweden | Sea trout | *Salmo trutta* | 252 | 0.60 | 9 | 10 | 9 | Cheung et al 2013 | F |
| United Kingdom | Rainbow trout | *Oncorhynchus mykiss* | 12800 | 0.36 | 6 | 12 | 9 | Cheung et al 2013 | F |
| United Kingdom | Sea trout | *Salmo trutta* | 520 | 0.60 | 9 | 10 | 9 | Cheung et al 2013 | F |

**References in Table 1**

Bolton, J.J. and Lüning, K. 1982. Optimal growth and maximal survival temperatures of Atlantic Laminaria Species (Phaeophyta) I culture. Marine Biology, 66:89-94

Cunjak, R.A. and Green, J. M. 1986. Influence of water temperature on behavioural interactions between juvenile brook charr, *Salvelinus fontinalis*, and rain bow trout, *Salmo gairdneri*. Journal of Canadian Zoology, 64:1288-1291.

Kneprath, W. G. and Meade, J.F. 1968. The effect of the environmental temperature on the fatty acid composition on the in vivo incoporation of 1-14C-Acetate in Goldfish (Carassius auratus L.). Lipids, 3:121-128.

Newel, R. I. E. 1980. Seasonal changes in the physiology, reproductive condition and carbohydrate content of the cockle *Cardium (=Cerastoderma) edule* (Bivalvia:Cardiidae). Marine Biology, 56:11-19.

Tank, M.W.T., Booms, G.H.R., Eding E.H., Wendelaar Bonga, S.E., Komen, J. 2000. Cold shocks: a stressor for common carp. Journal of Fish Biology, 57:881-894.
